# Detection of *Clostridium botulinum* in Some Egyptian Fish Products, Its Control In Vitro Using *Citrus* Leaves Extracts, and Applicability of *Citrus limon* Leaf Extract in Tuna

**DOI:** 10.3390/foods12071466

**Published:** 2023-03-29

**Authors:** Gamal Hamad, Elsayed E. Hafez, Sherien E. Sobhy, Taha Mehany, Reham A. Elfayoumy, Eman M. Elghazaly, Michael Eskander, Rasha G. Tawfik, Saleh M. Hussein, Leonel Pereira

**Affiliations:** 1 Department of Food Technology, Arid Lands Cultivation Research Institute, City of Scientific Research and Technological Applications, New Borg El-Arab 21934, Egypt; 2 Department of Plant Protection and Biomolecular Diagnosis, Arid Lands Cultivation Research Institute, City of Scientific Research and Technology Applications, New Borg El-Arab 21934, Egypt; 3 Department of Botany and Microbiology, Faculty of Science, Damietta University, Damietta 34511, Egypt; 4 Department of Microbiology, Faculty of Veterinary Medicine, Matrouh University, Matrouh 51511, Egypt; 5 Department of Food Hygiene and Control, Faculty of Veterinary Medicine, Alexandria University, Alexandria 22758, Egypt; 6 Department of Microbiology, Faculty of Veterinary Medicine, Alexandria University, Alexandria 22758, Egypt; 7 Department of Food Science and Technology, Faculty of Agriculture, Al-Azhar University, Assiut 71524, Egypt; 8 MARE–Marine and Environmental Sciences Centre/ARNET–Aquatic Research Network, Department of Life Sciences, University of Coimbra, Calçada Martim de Freitas, 3000-456 Coimbra, Portugal

**Keywords:** *Clostridium botulinum*, natural antioxidants, multiplex PCR, fishery products, *Citrus*, natural antimicrobials, natural extracts, shelf-life, botulism sickness

## Abstract

This study aims to detect *Clostridium botulinum* and its control using natural leaf extracts of *Citrus limon*, *Citrus sinensis*, and *Citrus unshiu* in Egyptian fish products, e.g., canned tuna, canned sardine, canned mackerel, fesikh, moloha, and renga, as well the application of *C. limon* in tuna. Moreover, the antibacterial activity of the *C. limon* leaf extract was also estimated. In the water extract, ascorbic acid, total flavonoid content (TFC), and total phenolic content (TPC) were determined by volumetric, aluminum chloride, and Folin–Ciocalteu approaches, respectively. The antioxidant ability of the extract was analyzed in vitro via free radical scavenging (DPPH) and Ferric reducing assays. The results showed variability in the distribution of the total number of positive *C. botulinum* in fish samples from three different governorates under study, which were (24) Alexandria, (16) Beheira, and (17) Gharbia, out of the 120 tested samples in each governorate. Additionally, the findings revealed that all three *Citrus* extracts contain an appropriate number of secondary metabolites, with a sustainable presence of saponin and tannins in the *C. limon* extract. Furthermore, all *Citrus* extracts inhibited bacterial growth by increasing the inhibition zone, with *C. limon* being the best extract (25 mm) compared to *C. sinensis* and *C. unshiu*. The overall results showed the high antioxidant and anti-*Clostridium* powers (*p* < 0.05) of *C. limon* leaf extract, indicating its preservative activity in fishery products during storage. Finally, *C. limon* leaf extract can fight off *C. botulinum* and is considered a promising natural preservation candidate in ensuring safe and fresh fishery products.

## 1. Introduction

Fishery products are proposed for consumption as a way to reduce human cardiovascular and other ailments, in addition to meeting the needs of a sizeable section of the Egyptian population. Moreover, they have significantly increased in number over the past few decades in various nations [1]. Many fish products (including smoked and salted fish) are high in protein, iodine, selenium, amino acid, mineral, lipid, and water-soluble vitamin compositions [2]. Further, the main nutritional benefit of processed fish products is their composition of extremely healthy fatty acids, which gives them nutritional properties [3].

This industry faces a strong neurotoxin produced by Gram-positive, obligately anaerobic, and spore-forming bacteria, such as *Clostridium botulinum* [4]. *C. botulinum* is widespread in numerous climatical regions under several environmental conditions, e.g., water bodies, lakes, soils, oceans, seas, and rivers [5], along with its prevalence in the gastrointestinal tract (GIT) of fish, foods, and animals [6]. The formation of spores, the main and frequent problem of these bacteria, offers them the opportunity of long-term maintenance in the environment until the beginning of satisfactory situations for growth [7]. Because *C. botulinum* strains may produce highly heat-resistant spores, they are the pathogen of greatest concern in low-acid canned food [8]. *C. botulinum* can produce highly effective neurotoxins, and there are numerous neurotoxin types known (A, B, C, D, E, F, G, HA, and X) that can cause foodborne botulism when they enter the food chain. Botulism has significant fatality rates in both people and animals. Human botulism is brought on by the different toxin types, A, B, E, and F, out of all the toxin types [9].

Botulism sickness, which affects a wide range of people, particularly newborns, can result in several issues and symptoms, including drooping eyes, drowsiness, nausea, vomiting, abdominal pains, difficulty swallowing or speaking, and paralysis [4]. Furthermore, paralysis of the diaphragm, throat, and many upper-airway muscles results in death [10] due to several factors, including the bacteria’s high potency, neuro-specificity, simplicity of production, and long-time availability. The dependence on molecular techniques has been widely used as they are conducted faster and give more reliable results with high-resolution visions of the structure and diversity of bacterial communities [11]. This approach has played a vital role in identifying many microorganisms and improving our understanding of the level of microbial complexity. As an effective method, the polymerase chain reaction (PCR) is utilized to identify and detect *C. botulinum*. Multiplex PCR is a highly sensitive method and is specific to the target genes in many pathogens, even in low DNA concentrations [12].

Nowadays, microorganisms are resistant to many antibiotics, and their tendency to use natural extracts has increased due to their biologically active compounds [13]. Additives have been approved in food industries due to their antimicrobial and antioxidant properties. These properties can increase the storage period by postponing oxidation and rancidity and preventing off-flavors in fish. The *Citrus* genus belongs to the Rutaceae family and includes forty species scattered globally. Indeed, citrus is one of the most vital fruits consumed either fresh and/or as a beverage owing to its pleasant flavor and high nutritive value. The antimicrobial, anticancer, antioxidant, anti-inflammatory, hepato-regenerating, and cardio-protective activities of *C. limon* extracts have been investigated [14].

*Citrus* leaves have a wide range of therapeutic potentials thanks to their flavonoids and limonoids, which display anticancer anti-inflammation effects [15]. In another research, *Citrus* leaves extracts demonstrated the strongest radical scavenging activity due to their flavonoid contents, including rutin, quercetin, apigenin, kaempferol, and nobiletin. These results suggest the potential utilization of *Citrus* leaves as a food supplement for human health [16]. Moreover, *C. limon* and its extracts have been classified as safe products according to the FDA [17]. *Citrus* extracts’ utility in food manufacturing is mainly in reducing/inhibiting spoilage, preserving the best quality, increasing safety, and showing strong antimicrobial activity checked against microorganisms, like *Pseudomonas aeruginosa*, *Salmonella typhimurium*, and *Micrococcus aureus* [13]; hence, it can be safely used as a food preservative.

Therefore, the current study aimed to (I) identify which fishery products might pose a risk of *C. botulinum*, (II) detect the toxin type that was most prevalent in the samples and tissues of commercial fish from the three investigated Egyptian governorates, and finally, (III) assess which *Citrus* (*C. limon*, *C. sinensis*, or *C. unshiu*) leaf extract has the most powerful effect on *C. botulinum* and can be used as a natural preservative.

## 2. Materials and Methods

### 2.1. Fishery Samples Collection

A total number of 360 fish samples were collected from three Egyptian governorates, Alexandria, Beheira, and Gharbia; each governorate comprises 120 samples collected from six fish products (i.e., canned tuna, canned sardine, canned mackerel, fesikh, moloha, and Renga), and 20 samples of each product. These samples were randomly collected from local supermarkets and fishery shops in 2022.

### 2.2. Fishery Samples Preparation

All fishery samples were refrigerated until they were tested. The surfaces of the cans were cleaned and dried, and the top surfaces were covered with ethanol (96%) and left to stand for 2 min until the ethanol was evaporated. The cans were opened with a sterile can opener and put in large plastic bags to avoid the spread of aerosols. Furthermore, 20 g of each fish was aseptically placed in a sterile mortar with 10 mL of sterile peptone water (0.1%) and then blended for 2 min. Amounts of 2 g of the prepared solid samples were inoculated into two screw-capped bottles; one contained 15 mL of the Trypticase peptone glucose yeast broth (TPGY; purchased from Oxoid Ltd., Basingstoke, Hampshire, UK) while the other contained 15 mL of a cooked meat medium (CMM; obtained from Oxoid Ltd., Basingstoke, Hampshire, UK). The inoculated media were incubated under anaerobic conditions for 7–10 days using an AnaeroGen^TM^ gas-generating kit (bought from Oxoid Ltd., Basingstoke, Hampshire, UK). The Trypticase peptone glucose yeast broth was incubated at 30 °C, overnight (16 h), to obtain subcultures. Then, the bacterial suspension of this overnight culture that was enriched on the TPGY medium was used for *C. botulinum* isolation and identification using Multiplex PCR (Agilent Technologies, SureCycler 8800 Thermal Cycler, Santa Clara, CA, USA), as well for DNA extraction.

### 2.3. Isolation and Identification of C. botulinum Using Conventional Methods

#### 2.3.1. Isolation of Pure Culture of *C. botulinum*

The isolation of *C. botulinum* in pure culture was enhanced by adding an equal volume of ethanol to 2 mL of enrichment culture. Then, the mixture was incubated at room temperature for 1 h. A loop of the enrichment culture of each sample was streaked on 2 plates of clostridial agar (obtained from HiMedia Laboratories GmbH, Einhausen, Germany). One of the streaked plates was incubated at 37 °C to isolate the proteolytic strains of *C. botulinum*, while the second plate was incubated at 28 °C to isolate the non-proteolytic strains of *C. botulinum*. Furthermore, the streaked plates were incubated under anaerobic conditions for 3–5 days by an AnaeroGen^TM^ gas-generating kit (purchased from Oxoid Ltd., Basingstoke, Hampshire, UK). After 3–5 days, all cultured plates were inspected for typical colonies of *C. botulinum*.

#### 2.3.2. Morphological Identification of *C. botulinum* Isolates

Morphological identification, microscopic examination, and the determination of catalase, lipase, and proteolytic activities were carried out according to Douillard et al. [18].

### 2.4. Molecular Characterization

#### 2.4.1. Bacterial DNA Extraction

Broth cultures of *C. botulinum* were centrifuged at 12.500× *g*/5 min, and the pellet was washed twofold with phosphate-buffered saline (PBS). The DNeasy^®^ blood and tissue kit (Qiagen, Venlo, Netherlands) was used for DNA isolation following the method for Gram-positive bacteria (as described in the instruction manual). DNA was kept at −20 °C until its use in the multiplex PCR.

#### 2.4.2. Multiplex PCR Components and Program

The genotyping of *C. botulinum* neurotoxin types (A, B, E, and F) and their detection were carried out using 4 pairs of primers. The sequences was proven in study of De Medici et al. [19] that used for detection and genotyping of *C. botulinum* neurotoxin type (A, B, E, and F) genes. The reaction mixture of the multiplex PCR (50 μL) contained 25 μL of 2× Multiplex PCR master mix (purchased from Qiagen, Spain), 2 μL of 0.3 μM of each primer (obtained from Bioneer, Daejeon, Republic of Korea), and 3 μL of purified DNA template and the volume was completed up to 50 μL using sterile dH_2_O [19].

The amplification was performed in a programmable thermal cycler (Biometra, ND, USA). The reaction conditions were initiated by a hot start at 95 °C/15 min, then they were followed by 35 cycles, each one comprising denaturation at 95 °C/30 s, further annealing at 51 °C/30 s, and extension at 72 °C/90 s, and finally, the reaction ended by a final extension at 72 °C/7 min.

#### 2.4.3. Detection of Multiplex PCR Products by Agarose Gel Electrophoresis

PCR end products were analyzed by 1.5% agarose gel electrophoresis in the presence of a standard DNA ladder (100 bp Nippon genetics) to measure the size of product bands and then visualized under an ultraviolet transilluminator.

### 2.5. Plant Samples Collection and Extracts Preparation

The leaves of *Citrus limon* (lemon), *Citrus sinensis* (orange), and *Citrus unshiu* (satsuma mandarin) were collected from the Experimental Farm of City of Scientific Research and Technological Applications (SRTA City), New Borg El Arab city, Egypt. The plant leaves were shade-dried for 3 days, successively ground to a fine powder using a blender, extracted in deionized H_2_O (1:20 *w*/*v*), centrifuged (ThermoFisher Scientific Co., Waltham, MA, USA) at 3000× *g*/15 min, and then filtrated. Aqueous leaf extracts were lyophilized by a vacuum freeze dryer (Lyophilizer, Model FDF 0350, Yangzhong, China) and stored for further analysis.

### 2.6. Phytochemical Screening of Citrus Leaf Extracts

Antioxidant compounds were determined in each leaf extract, as prescribed by Sobhy et al. [20], to assess their biological effect. Secondary metabolites, e.g., saponin and tannins, were estimated quantitatively [21,22]. In addition to secondary metabolites, antioxidant compounds, such as flavonoids, phenolics, and ascorbate, were assayed following the methods of Chang et al. [23]. Total antioxidant capacity, by DPPH and phosphomolybdate assay (PMA), was determined according to the methods of El Sohaimy et al. [24]. Moreover, Ferric reducing antioxidant power was evaluated following the method of Saeed et al. [25].

### 2.7. Determination of Antibacterial Activity of Citrus Leaves Extracts and Their Minimum Inhibitory Concentration (MIC)

The wild microbial strains were grown in a nutrient broth at 37 °C/24 h, and the *C. botulinum* suspension of grown cultures was prepared and adjusted to a density of 10^6^ colony-forming units (CFU)/mL, and then spread on MHM plates. After dryness, three Citrus leaf extracts (*C. limon*, *C. sinensis*, and *C. unshiu*) were loaded onto each separate disk (20 µL were taken from 100 mg/mL from each leaf extract concentration), and the plates were maintained at 4 °C/30 min and then incubated at 37 °C/24 h. The clear inhibitory zones obtained were recorded in mm, considering the anti-*C. botulinum* activity of these Citrus leaf extracts [26]. Moreover, a set of 7 concentrations of reconstituted aqueous leaf extracts, i.e., 1.56, 3.1, 6.25, 12.5, 25, 50, and 100 mg/mL, were examined to determine the minimum inhibitory concentration (MIC) of *C. limon*, *C. sinensis*, and *C. unshiu* leaf extracts against pathogenic strains (*C. botulinum*) [26].

### 2.8. Storage Study and Shelf Life of Tuna Supplemented with of C. limon Leaf Extracts

The fishes were prepared as fillets weighing approximately 100–150 g. After that, the fillets were assigned into five groups: (1) the control group (untreated with an extract), (2) control tuna meat, which was dipped in chilled distilled water for 20 min, and groups 3, 4, and 5, which were infected with a *C. botulinum* strain (10^6^ CFU/mL) and treated by being dipped in *C. limon* leaf extract at concentrations of 10%, 20%, and 30%, respectively, for 20 min. After dipping, the chunks were drained at ambient temperature for 3 min. The fillets were placed in sterile polythene bags and stored at 4 °C. The samples were randomly removed from each treatment to assess the preservative effect of *C. limon* leaf extracts on the shelf-life of tuna fillets under several storage periods, i.e., 0, 2, 4, 6, 8, 10, and 12 days. The samples representing all regions of the chunks of the respective lots (in correct quantities) were weighed and transferred for microbiological analysis at every 2-day interval [27]. For the purpose of microbial analyses, the cooled samples were homogenized for 1 min and then incubated at 37 °C/24 h in a CO*_2_* incubator. An amount of 1 mL was added to 9 mL of the peptone broth and incubated at 37 °C/24 h in a CO*_2_* incubator. The total anaerobic plate counts were taken on TPGY agar under anaerobic conditions.

### 2.9. Sensory Evaluation

The tuna samples were maintained at room temperature, 25 °C/10 min, prior to assessment. The panelists evaluated the tuna sensorial attributes for both the control and samples treated with different concentrations (10%, 20%, and 30%) of *C. limon* leaf extract based on the following criteria: odor, taste, color, texture, and overall acceptability (10 points each item), with a scale ranging from 1 to 9, where 9 = excellent, 8 = very good, 7 = very good, 6 = good, 5 = medium, 4 = fair, 3 = poor, 2 = very poor, and 1 = very, very poor, as described by Hamad et al. [28].

### 2.10. Statistical Analysis

The obtained results were statistically analyzed based on the SPSS software (version 23, IBM SPSS Statistics for Windows, IBM Corp., New York, NY, USA) using a one-way analysis of variance (ANOVA) to determine the degree of significance for the obtained variations of the used treatments. The expressed data were the mean of three the replicates ± the standard deviation, and the significant level was estimated at *p* < 0.05.

## 3. Results and Discussion

### 3.1. Detection of C. botulinum Types (A, B, E and F) in Fishery Product Samples from Three Governorates

**Figure 1:**
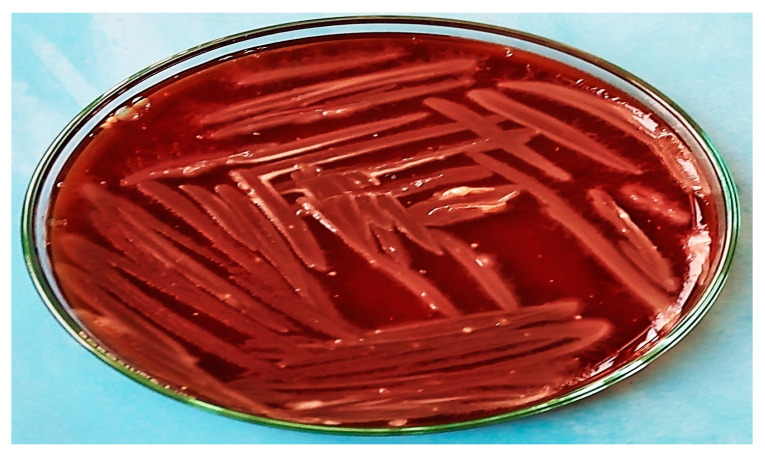
Blood agar media inoculated with *C. botulinum* showing a narrow zone of β-hemolysis surrounding the colonies.

**Figure 2:**
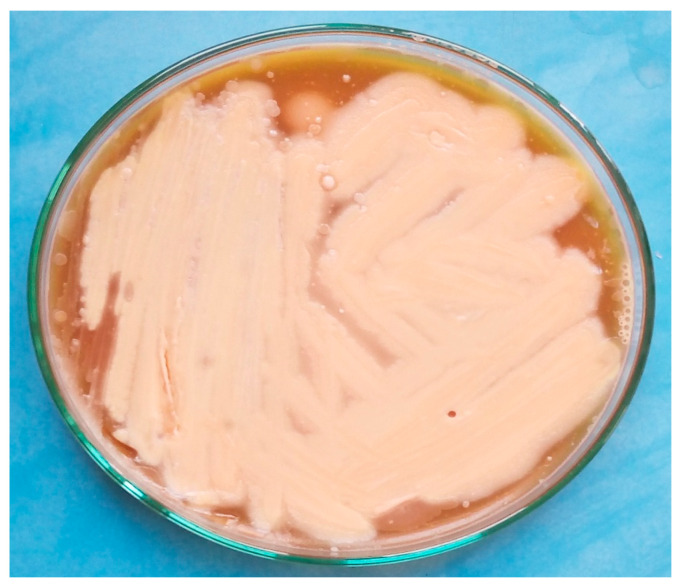
Anaerobic egg yolk medium inoculated with *C. botulinum* showing lipase-positive colonies.

The collected data showed typical characteristics of *C. botulinum*, such as being Gram-positive, anaerobic, and large, having a smooth surface, being grey in color, and being straight or marginally curved with oval-like subterminal spores. Films stained with Gram stain were prepared from the isolated colonies. The activity of the β-hemolysis enzyme of this bacterium on blood agar containing 10% sheep blood showed narrow zones, as shown in Figure 1. On the other hand, catalase enzymes revealed negative results, while there were positive findings with lipase enzymes (Figure 2).

According to the data obtained from the Alexandria governorate in Table 1, 24 of 120 examined samples tested positive for *C. botulinum*. Canned sardine showed the highest percentage of positive isolates (35%), followed by canned tuna (25%), while moloha had the lowest percentage (10%), with an abundance of type E (35%) being found in all isolated samples with a high prevalence in fesikh and moloha (10%).

In the same regard, Table 2 represents the results of the Beheira governorate, which postulated that 16 samples of 120 examined were positive. Renga samples possessed the highest percentage of positive isolates (20%), followed by fesikh, canned tuna, and mackerel (15%). The most abundant *C. botulinum* toxin type was type E, and the percentage reached 60% in all tested products.

In the Gharbia governorate, the detection of *C. botulinum* types revealed that from the 120 studied samples of the six products under study, only 17 samples were positive. Canned sardines had the highest level of isolated bacterial samples (25%), followed by canned mackerel and tuna (20%), while those of renga, fesikh, and moloha were considered low with percentages of 15%, 10%, and 5%, respectively. Further, the type E toxin was the most prevalent type with a total count of 45% among all tested types, whereas type F was completely absent (Table 3).

These results are in agreement with those of Hamad et al. [28] regarding the majority of toxic microorganisms in some Egyptian canned foods, including fish products, that demonstrated the number of isolated strains, chiefly *C. botulinum* type E, which may cause the potential threat of infant botulism and foodborne botulism. Botulism is a lethal neuroparalytic disease resulting from the action of a highly potent neurotoxin formed during the maturation of *C. botulinum*. Foodborne botulism is a public health predicament due to its cruelty and imminent epidemic spread. *C. botulinum*-contaminated canned fish, meat, and vegetables may produce toxins that are heat sensitive and not properly handled [29]. The production of such toxins could be caused by certain circumstances, such as processed and non-processed food spoilage with either vegetative bacteria, cells, or spores. Insufficient treatment is enough to suppress spore germination and/or recontamination after food processing. A high yield of bacterial toxins is produced through anaerobic conditions that are suitable for bacterial spore germination [30]. The prevalence of the type E toxin in fish and fishery products is important because it grows at low temperatures and is associated with low spoilage [31]. In the same regard, Hyytiä et al. [32] investigated the need to clean, wrap, and disembowel fish as well as the possibility of a risk. This risk exists due to the adherence of *C. botulinum* spores on the surface of fishes which contaminate the muscle tissue during processing. Muscles under the fish surface could save the anaerobic conditions suitable for the growth of vegetative cells and affect toxin production. The present study revealed that the major positive fish products were contaminated with *C. botulinum*, and the high occurrence of *C. botulinum* in the examined fishery products may warrant their qualification as unhygienic products.

Generally, *C. botulinum* strains can release their neurotoxins in foods under favorable conditions, which include contaminated foods with bacterial spores, inadequate heat treatment, recontamination following processing and anaerobic canned products [28]. This could explain the clostridial form difference among the fishery items studied and reported in this study.

### 3.2. Genotyping of Toxin Types Using Multiplex PCR

**Figure 3:**
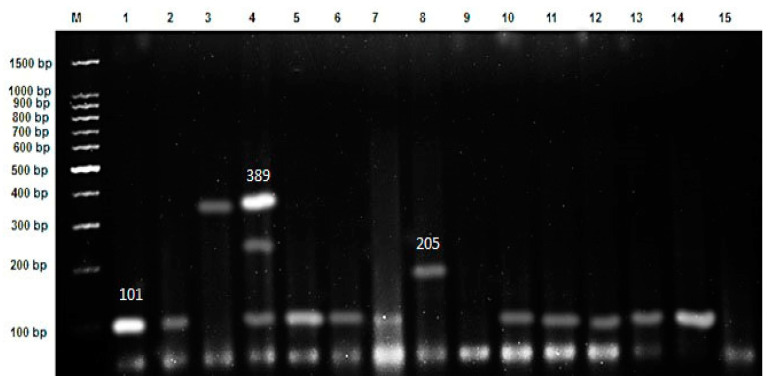
Ethidium bromide-stained 1.5% agarose gel with multiplex PCR products showed *C. botulinum* neurotoxin genes from 15 bacterial isolates from the Alexandria governorate (lanes 1–15), where M represents a 1500 bp DNA ladder.

**Figure 4:**
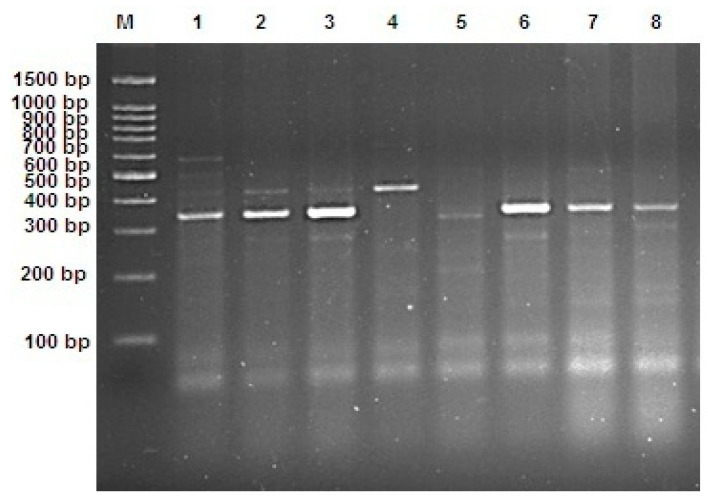
Ethidium bromide-stained 1.5% agarose gel with multiplex PCR products showed *C. botulinum* neurotoxin genes from eight bacterial samples isolated from the Beheira governorate (lanes 1–8), where M represents a 1500 bp DNA ladder.

**Figure 5:**
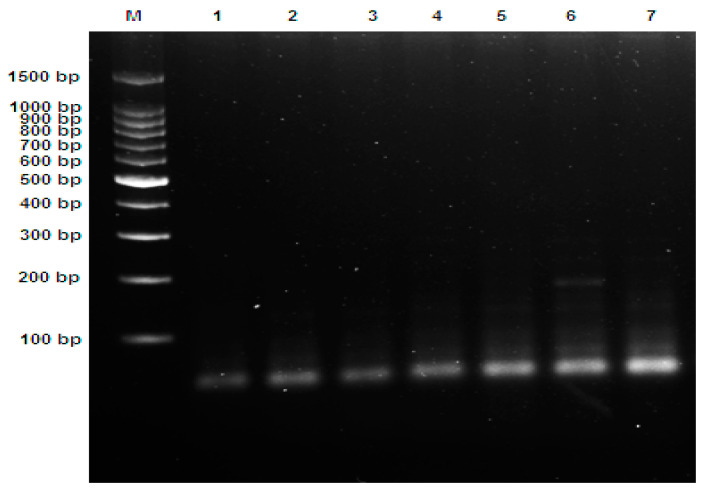
Ethidium bromide-stained 1.5% agarose gel with multiplex PCR products showed *C. botulinum* neurotoxin genes from seven bacterial isolates from the Gharbia governorate (lanes 1–7), where M represents a 1500 bp DNA ladder.

De Medici et al. [19] showed the neurotoxin genes obtained by the multiplex PCR, which confirm the presence/absence of neurotoxin genes produced by *C. botulinum* in the tested fishery products in the current study. The multiplex PCR yielded the expected differences of PCR products, which grouped the examined samples with *C. botulinum* into the types A, B, E, and F. The molecular sizes of the obtained genotypes were 101 bp for type A, 205 bp for type B, and 389 bp for type E, and a band with a molecular size of 543 bp was observed for type F. Moreover, The PCR band patterns of the examined samples were illustrated in Figure 3, Figure 4 and Figure 5 and these results agree with those reported by Herman et al. [33].

The data presented in Table 4 and Figure 3 regarding the Alexandria governorate postulates that clear bands were formed at 101, 205, and 389 bp for clostridial strain types A, B, and E, respectively. The bacterial isolates were divided into four main categories that differ in their profile pattern. The first comprises type A only, the second is characterized by type E, the third group contains type B, and the final group contains a mix of three toxin types, A, B, and E.

Regarding the Beheira governorate data presented in Table 5 and Figure 4, the results reveal that eight isolated samples were separated into four main groups; three groups had combined toxin types, E and F (1), A, B, and E (5), and A and E (6, 7) in addition to the separate toxin type E in samples 2, 3, 8.

The data presented in Table 6 and Figure 5 display the Gharbia governorate results; in the clear bands, different toxin types were formed at 101 and 205 bp for types A and B, respectively, and the sets of this governate were divided into two main groups: one contained a single toxin, type A (1, 2, 3, 4, and 5), and the other group contained two toxin types, A and B (6 and 7).

It can be concluded that multiplex PCR is considered a good procedure when compared with the other traditional methods because it is a rapid and sensitive detection method. Furthermore, the multiplex PCR employed its specificity based on toxin type-specific primers [34]. More than 167 million tons of fishery products are produced annually, according to FAO [35]; however, about 87.5% of these products are consumed by humans. For canned fish, about 19 million tons are produced, which represents 13% of the world’s total production. Many types of fish are employed in canning production, such as tuna, sardines, and mackerels [36]. The data mentioned above exhibits that canned sardine was the most contaminated among the examined samples. These high contamination percentages may be due to the misconduct in preserving such products [37] and the existence of a high copy number of spoilage bacteria. Consequently, it was observed that thermal treatment could be the most powerful method for inhibiting the spoilage bacteria in sardines.

Additionally, the chilling of crude materials during the storage time should be performed before production. The nature of the crude material, whether contaminated or not, can be tested if any changes occur in capacity [38]. The results obtained in this study are in accordance with those obtained by Fleck-Derderian et al. [39], in which they confirmed the prevalence of *C. botulinum*, especially type E, in different fish products. The prevalence of *C. botulinum* was, in percentages, up to 17% among the 197 foodborne outbreaks caused between 1920 and 2014.

### 3.3. Plant Extract Phytochemical Screening

The data reveal that all citrus extracts contain an appropriate number of secondary metabolites, with the sustained presence of saponin and tannins in *C. limon* extract (Table 7). Furthermore, flavonoids, the most important natural antioxidant with broad-spectrum chemical and biological properties including radical scavenging properties, are commonly found in Citrus species, which in turn are a rich source of hesperidin (a member of the flavanone group of flavonoids). Abd Ghafar et al. [40] postulated that this plant extract contains different active materials, which exhibit a wide range of properties, including antioxidant, anticarcinogenic, antihypotensive, and antimicrobial properties. It was observed that ascorbic acid and vitamin C (essential secondary metabolites for human health) are the most prominent antioxidant phytochemicals in *C. limon* extract. The importance of ascorbic acid as an antioxidant may be attributed to its ability to neutralize reactive oxygen species and inhibit the production of free radicals [41]. Moreover, it has a significant impact on the immune system, regulates the reduction of inflammatory mediators and macrophage activity, and, in high concentrations, can potentially have a bacteriostatic effect [42].

The free radical scavenging activity of citrus extracts was determined by a DPPH free radical scavenging assay. Highly effective free radical scavenging was observed in the *C. limon* extract compared to *C. sinensis* and *C. unshiu*. Moreover, the Ferric reducing assay was employed to determine the reducing potential of the tested extracts. The highest reducing potential was shown in the *C. limon* extract (Table 7), with a high total antioxidant capacity being symbolized by the PMA.

### 3.4. Antibacterial Activity of Citrus Leaf Plant Extract

**Figure 6:**
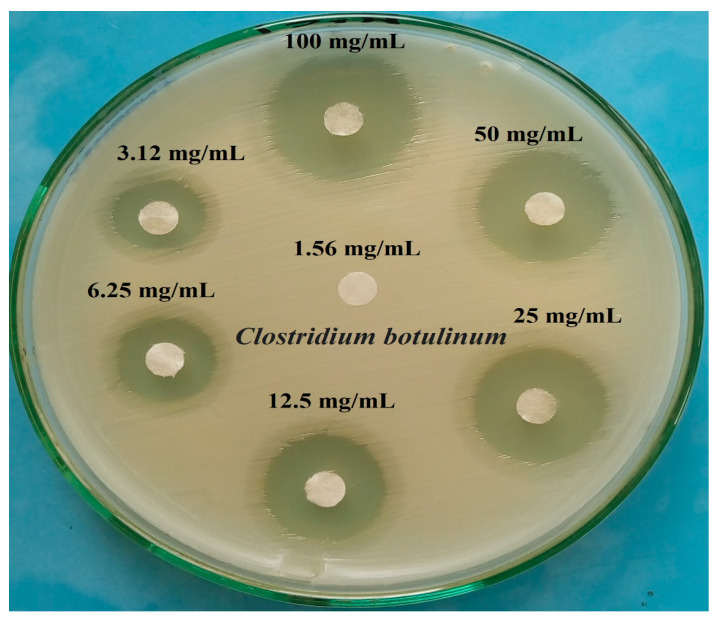
Minimum inhibitory concentrations (MICs) of *Citrus limon* leaf extract against *C. botulinum* type E using an agar disk diffusion assay.

As presented in Table 8 and Figure 6, *C. botulinum* pathogenic bacteria were tested to evaluate the antimicrobial activity of *C. limon*, *C. sinensis*, and *C. unshiu* leaf extracts. The results revealed that all the *Citrus* extracts significantly inhibited bacterial growth by increasing the inhibition zone, while *C. limon* was the best extract (25 mm) when compared to *C. sinensis* and *C. unshiu*. In addition, the synergistic antimicrobial effects of *Combretum hereroense* leaf extract in combination with leaf extracts of the *Citrus lemon* and *Apodytes dimidiata* (Metteniusaceae) species were investigated against *Mycobacterium smegmatis* via a microdilution approach. This study reported the potent antimycobacteria potential of sub-fractions of *A. dimidiata* against the MDR-TB field strain. This reflects the possibility of isolating some active compounds that can be used to counter resistant strains of *tuberculosis* [43]. Moreover, multi-species-containing herbal medications could have good antimicrobial potency. Thus, natural plant extracts and their accompanying bioactive substances are known to potentiate the effects of antibiotics [44].

The potential biological activity observed in *C. limon* may be accredited to the enriched levels of bioactive antioxidant compounds. Some of these phytochemicals are ascorbic acid, tannins, saponins, flavonoids, and phenolics, with high antioxidant capacity and reduced power activity. Moreover, *C. limon* extract exhibited pronounced inhibitory activity towards *C. botulinum*, showing a positive correlation with the observed antimicrobial activity provided by flavonoids and phenolics. These results indicated that *C. limon* leaf extracts might be used as an alternative to synthetic antibacterial agents, with great potential for application as an environmentally friendly, tasty preservative in food industries.

### 3.5. Storage Study, Shelf Life, and Sensory Evaluation of Tuna Supplemented with C. limon Extract

Data presented in Table 9 reveal that the usage of *C. limon* extract in storage decreased the total count of bacteria by increasing the storage period until it disappeared. The metabolomic profile of *C. limon* extract investigated by a recent study showed that *C. limon* leaves contain 26 different organic acids and their derivatives (benzoic acid, ferulic acid, and fumaric acid), 21 amino acids (alanine, asparagine, and glutamine), and 13 sugars and sugar alcohols (fructose, galactose, and maltose,). All these active components may have a role in giving *C. limon* the importance of being a good preservative [42].

Moreover, the use of *C. limon* leaf extract as a natural preservative resulted in the maintained quality of tuna meat samples when tested by ten different persons, and in the preserved color, odor, taste, texture, and overall acceptance (Table 10).

## 4. Conclusions

This study reported the high incidence of *C. botulinum* in all tested fishery products from three different Egyptian governorates, which is considered one of the main biological threats that cause foodborne pathogenesis. This study highlights the control of *C. botulinum* (a) in contaminated fish products using the leaf extracts of *Citrus limon*, *Citrus sinensis,* and *Citrus unshiu* as control agents. The current findings demonstrate the antioxidant activities of *Citrus* leaf extracts. In addition to their broad potential to suppress the growth of *C. botulinum,* these bioactive components can be used to treat bacterial contamination and as preservatives.

In addition, all *Citrus* extracts inhibited *C. botulinum* growth by increasing the inhibition zone, with *C. limon* being the most potent extract, followed by *C. sinensis* and *C. unshiu*. Overall, the high antioxidant and anti-*Clostridium* powers of *C. limon* leaf extract, which indicate its preservative activity in fishery products during storage, can be concluded. *C. limon* leaf extract has the potential to prevent *C. botulinum* growth and serve as a promising natural preservative agent for keeping fishery products fresh and safe. Further investigations should be carried out into the potential toxicity of *Citrus* leaf extracts at high concentrations in both in vitro and in vivo studies.

## Figures and Tables

**Table 1:** Detection of *C. botulinum* types (A, B, E, and F) in fish product samples from the Alexandria governorate.

Types of Fish Products	Total No. of Analyzed Samples	Total Bacterial Isolates		*C. botulinum* Type							
				A		B		E		F	
		No.	%	No.	%	No.	%	No.	%	No.	%
Canned tuna	20	5	25	3	15	1	5	1	5	0	0
Canned sardine		7	35	5	25	0	0	1	5	1	5
Canned mackerel		3	15	1	5	1	5	0	0	1	5
Fesikh		3	15	0	0	1	5	2	10	0	0
Moloha		2	10	0	0	0	0	2	10	0	0
Renga		4	20	0	0	2	10	2	10	0	0

**Table 2:** Detection of *C. botulinum* types (A, B, E, and F) in fish product samples from the Beheira governorate.

Types of Fish Products	Total No. of Analyzed Samples	Total Bacterial Isolates		*C. botulinum* Type							
				A		B		E		F	
		No.	%	No.	%	No.	%	No.	%	No.	%
Canned tuna	20	3	15	0	0	0	0	3	15	0	0
Canned sardines		1	5	0	0	0	0	1	5	0	0
Canned mackerel		3	15	0	0	0	0	3	15	0	0
Fesikh		3	15	0	0	1	5	2	10	0	0
Moloha		2	10	0	0	0	0	2	10	0	0
Renga		4	20	2	10	0	0	2	10	0	0

**Table 3:** Detection of *C. botulinum* types (A, B, E, and F) in fish product samples from the Gharbia governorate.

Types of Fish Products	Total No. of Analyzed Samples	Total Bacterial Isolates		*C. botulinum* Type							
				A		B		E		F	
		No.	%	No.	%	No.	%	No.	%	No.	%
Canned tuna	20	2	20	0	0	2	10	0	0	0	0
Canned sardines		5	25	1	5	1	5	3	15	0	0
Canned mackerel		2	20	1	5	0	0	1	5	0	0
Fesikh		4	10	0	0	2	10	2	10	0	0
Moloha		1	5	0	0	0	0	1	5	0	0
Renga		3	15	0	0	0	0	3	15	0	0

**Table 4:** The banding pattern for the 15 studied bacterial isolates (isolated from the Alexandria governorate) using multiplex PCR.

No. of Bacterial Isolates	No. of Detected Bands	Length of Bands (bp)	Toxin Type
1	1	101	A
2	1	101	
3	1	389	E
4	3	389, 205, 101	A, B, E
5	1	101	A
6	1	101	
7	1	101	
8	1	205	B
9	1	205	
10	1	101	A
11	1	101	
12	1	101	
13	1	101	
14	3	389, 205, 101	A, B, E
15	1	101	A

**Table 5:** The banding pattern for the eight studied bacterial isolates (isolated from Beheira governorate) using multiplex PCR.

No. of Bacterial Isolates	No. of Detected Bands	Length of Bands (bp)	Toxin Type
1	2	389, 543	E, F
2	1	389	E
3	1	389	
4	0	--	
5	3	101, 205, 389	A, B, E
6	2	101, 389	A, E
7	2	101, 389	
8	1	389	E

**Table 6:** The banding pattern for the seven studied bacterial isolates (isolated from the Gharbia governorate) using multiplex PCR.

No. of Bacterial Isolates	No. of Detected Bands	Length of Bands (bp)	Toxin Type
1	1	101	A
2	1	101	
3	1	101	
4	1	101	
5	1	101	
6	2	101, 205	A, B
7	2	101, 205	

**Table 7:** Phytochemical screening of bioactive components of *C. limon*, *C. sinensis,* and *C. unshiu* leaf extracts.

Category	Parameter	*C. limon*	*C. sinensis*	*C. unshiu*
Secondary metabolites (mg/g DM)	Saponin	55.7 ± 3.4 ^a^	23.3 ± 2.8 ^b^	7.9 ± 1.2 ^c^
	Tannins	30.2 ± 3.8 ^a^	2.7 ± 1.1 ^c^	5.3 ± 1.5 ^b^
Antioxidant compounds (%)	Phenolic	146 ± 14.8 ^a^	99 ± 5.9 ^b^	31 ± 3.9 ^c^
	Flavonoids	68 ± 6.9 ^a^	18 ± 2.0 ^b^	11 ± 1.2 ^c^
	Ascorbate	35.7 ± 1.7 ^a^	26.4 ± 2.1 ^b^	18.5 ± 1.3 ^c^
Total antioxidant capacity (mg/g DM)	DPPH	51.0 ± 2.3 ^a^	26.3 ± 0.2 ^b^	10.3 ± 1.8 ^c^
	PMA	34.2 ± 3.4 ^a^	18.8 ± 1.7 ^b^	12.6 ± 1.2 ^c^
	Ferric reducing power	49.3 ± 0.2 ^a^	47.5 ± 0.9 ^b^	24.6 ± 0.5 ^c^

Data represented are the means of triplicates ± standards error of means. ^a, b,^ and ^c^: data in the same row followed by different superscript letters differ significantly (*p* < 0.05).

**Table 8:** Antibacterial activity and minimum inhibitory concentrations (MICs) of *C. limon*, *C. sinensis,* and *C. unshiu* leaf extracts against *C. botulinum* type E using an agar disk diffusion assay.

Plant Leaf Extracts	Concentration mg/mL	Inhibition Zone Diameter (mm)	
*Citrus limon* (lemon)	100	25 ± 2 ^a^	
*Citrus sinensis* (orange)		22 ± 1 ^bc^	
*Citrus unshiu* (mandarin)		23 ± 2 ^ab^	
Minimum inhibitory concentrations (MICs)			
Plant Extracts Conc. (mg/mL)	*C. limon*	*C. sinensis*	*C. unshiu*
100	25 ± 2 ^a^	22 ± 1 ^c^	23 ± 2 ^ab^
50	22 ± 1 ^a^	18 ± 2 ^c^	19 ± 2 ^b^
25	19 ± 3 ^a^	15 ± 1 ^c^	16 ± 1 ^b^
12.5	16 ± 2 ^a^	10 ± 2 ^c^	12 ± 2 ^b^
6.25	12 ± 2 ^a^	ND	9 ± 2 ^b^
3.12	8 ± 1 ^a^	ND	ND
1.56	ND	ND	ND

Data represented are the means of triplicates ± standards error of means. ^a, b,^ and ^c^: data in the same row followed by different superscript letters differ significantly (*p* < 0.05). ND: Not detected.

**Table 9:** Storage study and shelf-life evaluation of tuna meat during refrigerated storage after treatment under different concentrations (10%, 20%, and 30%) of limon leaf extract.

*C. limon* Extract Conc.	Total Anaerobic Plate Count per Day of Storage					
	0	2	4	6	8	12
Control (0%)	1 × 10^6^	1.7 × 10^6^	2.3 × 10^6^	2.8 × 10^6^	3.6 × 10^6^	4.5 × 10^6^
(10%)	1 × 10^6^	1.2 × 10^5^	0.9 × 10^4^	0.7 × 10^3^	0.5 × 10^2^	0.0
(20%)	1 × 10^6^	0.7 × 10^4^	0.6 × 10^3^	0.4 × 10^2^	30	0.0
(30%)	1 × 10^6^	0.5 × 10^3^	0.2 × 10^2^	45	0.0	0.0

**Table 10:** Sensory evaluation of tuna quality for control and treatment with different concentrations (10%, 20%, and 30%) of *C. limon* leaf extract.

Concentration of *C. limon* Leaf Extract in Tuna Samples	Color	Odor	Taste	Texture	Overall Acceptance
	10 Persons Each				
Control (0%)	8.1 ± 0.7 ^a^	7.8 ± 1.0 ^ab^	8.1 ± 0.7 ^a^	8.0 ± 0.8 ^a^	8.4 ± 0.5 ^a^
10%	8.5 ± 0.5 ^a^	8.1 ± 0.5 ^a^	8.7 ± 0.5 ^a^	8.5 ± 0.6 ^a^	8.4 ± 0.5 ^a^
20%	8.4 ± 0.6 ^a^	8.5 ± 0.4 ^a^	8.7 ± 0.3 ^a^	8.5 ± 0.4 ^a^	8.7 ± 0.3 ^a^
30%	7.7 ± 0.6 ^ab^	8.0 ± 0.8 ^a^	8.0 ± 0.8 ^a^	8.2 ± 0.6 ^a^	8.2 ± 0.4 ^a^

Data represented are the means of ten replicates ± standards error of means. ^a^ and ^b^: data in the same column followed by different superscript letters differ significantly (*p* < 0.05). Score system: 9 = Excellent, 8 = very, very good, 7 = very good, 6 = good, 5 = medium, 4 = fair, 3 = poor, 2 = very poor, 1 = very, very poor.

## Data Availability

The data presented in this study are available upon request from the corresponding author.
